# Chronic Protein Restriction in Mice Impacts Placental Function and Maternal Body Weight before Fetal Growth

**DOI:** 10.1371/journal.pone.0152227

**Published:** 2016-03-28

**Authors:** Paula N. Gonzalez, Malgorzata Gasperowicz, Jimena Barbeito-Andrés, Natasha Klenin, James C. Cross, Benedikt Hallgrímsson

**Affiliations:** 1 Instituto de Genética Veterinaria, CCT-CONICET, La Plata, Argentina; 2 de Ciencias Naturales y Museo, UNLP, La Plata, Argentina; 3 Department of Comparative Biology and Experimental Medicine, Faculty of Veterinary Medicine, and the Alberta Children’s Hospital Research Institute, University of Calgary, Calgary, Alberta, Canada; 4 Department Biochemistry & Molecular Biology, University of Calgary, Calgary, Alberta, Canada; 5 Department of Cell Biology and Anatomy, Alberta Children’s Hospital Research Institute, and McCaig Institute for Bone and Joint Health. University of Calgary, Calgary, Alberta, Canada; Medical Faculty, Otto-von-Guericke University Magdeburg, Medical Faculty, GERMANY

## Abstract

Mechanisms of resource allocation are essential for maternal and fetal survival, particularly when the availability of nutrients is limited. We investigated the responses of feto-placental development to maternal chronic protein malnutrition to test the hypothesis that maternal low protein diet produces differential growth restriction of placental and fetal tissues, and adaptive changes in the placenta that may mitigate impacts on fetal growth. C57BL/6J female mice were fed either a low-protein diet (6% protein) or control isocaloric diet (20% protein). On embryonic days E10.5, 17.5 and 18.5 tissue samples were prepared for morphometric, histological and quantitative RT-PCR analyses, which included markers of trophoblast cell subtypes. Potential endocrine adaptations were assessed by the expression of Prolactin-related hormone genes. In the low protein group, placenta weight was significantly lower at E10.5, followed by reduction of maternal weight at E17.5, while the fetuses became significantly lighter no earlier than at E18.5. Fetal head at E18.5 in the low protein group, though smaller than controls, was larger than expected for body size. The relative size and shape of the cranial vault and the flexion of the cranial base was affected by E17.5 and more severely by E18.5. The junctional zone, a placenta layer rich in endocrine and energy storing glycogen cells, was smaller in low protein placentas as well as the expression of *Pcdh12*, a marker of glycogen trophoblast cells. Placental hormone gene *Prl3a1* was altered in response to low protein diet: expression was elevated at E17.5 when fetuses were still growing normally, but dropped sharply by E18.5 in parallel with the slowing of fetal growth. This model suggests that nutrients are preferentially allocated to sustain fetal and brain growth and suggests the placenta as a nutrient sensor in early gestation with a role in mitigating impacts of poor maternal nutrition on fetal growth.

## Introduction

In humans, as in all eutherians, prenatal growth relies on the nutrient supply and gas exchange mediated by the placenta. The inadequate availability of nutrients and/or oxygen for the fetus due to environmental factors such as maternal malnutrition or deficient blood flow caused by placental insufficiency, frequently results in intrauterine growth restriction (IUGR) [1–3]. Although the resulting smaller size has been considered a relatively beneficial adjustment and an adaptive response under stressful conditions [4], IUGR is associated with higher rates of perinatal morbidity and mortality, as well as long term consequences such as obesity, cardiovascular disease or type II diabetes in human populations [5]. Consequently, there is great interest in understanding which changes occur in feto-placental development under stressful conditions and their adaptive role in sustaining fetal growth as well as the demand of energy for the growth and maintenance of the placenta [6, 7]. Animal models provide a valuable tool to gain insight into these questions and have been extensively used to evaluate the effect of different perturbations on prenatal development [8, 9]. Experiments in rodents support the hypothesis that maternal malnutrition does not affect each fetal tissue to the same extent. In humans as well as in animal models, brain weight and neurocranial size of neonates and adults are usually less affected by nutrient restriction than body weight and facial size, which suggest an adaptive “brain sparing” effect [10–13]. However, direct tests of in utero trade off between tissues are deficient and the available studies in humans usually measure overall fetal and placental weight only [14].

Experimental studies in rats and mice show that isocaloric low protein diets and reduced energy intake throughout gestation induce changes in placental weight and morphology [15–21]. These effects are highly variable depending on the IUGR model and the gestational age analyzed. Accordingly, it is observed that protein restriction during pregnancy can induce significant reduction in fetal and/or placental weight already at mid-gestation, or only by the very end of pregnancy [15, 16, 22–25]. Despite the intensive research in this area, most studies have focused on the effect of short term malnutrition, and thus, the effects of chronic malnutrition -i.e., starting before and continuing throughout gestation- which more closely resembles human conditions of poor nutrition, remain largely unexplored.

Of the three main compartments in the mouse placenta—maternal decidua, junctional zone and labyrinth—the labyrinth layer is the most studied due to its essential role in feto-maternal exchange of gases and nutrients [26, 27], while the junctional zone is still relatively poorly understood. Glycogen trophoblast cells, which are ubiquitous in the junctional zone, accumulate glucose and store it in the form of glycogen, which suggests a potential role of the junctional zone as an energy storing layer that may play an adaptive role [28–31]. In addition, trophoblast giant cells, spongiotrophoblast and glycogen trophoblast cells within the junctional zone express many hormones which in mice include 22 related to Prolactin [32–35], some of which are implicated as drivers of changes in maternal metabolism during pregnancy. The human placenta also produces several metabolic hormones and ones related to Prolactin and Growth Hormone are also associated with changes in maternal metabolism thought to promote nutrient availability for transport across the placenta [36].

This study examined feto-placental growth during mid to late gestation in a mouse model of long term maternal protein undernutrition. Insufficient protein intake and overreliance on carbohydrates is prevalent in low income countries as well as among vulnerable populations in middle and high income countries [37]. We hypothesized that exposure to chronic low protein diet during prenatal growth would result in a differential resource allocation among feto-placental tissues that is evidenced by an asymmetric growth impairment. Fetal and placental growth parameters were measured at three points in gestation: E10.5 and E17.5 represent the developing and mature placenta before and after the mid-gestation, and in which the labyrinth and junctional zone can be distinguished [38]. E17.5 and E18.5 were analyzed to register changes related to the rapid growth of the fetus by the end of gestation [39]. In particular, neurogenesis in most brain cell populations peaks between E9.5 (just after neural tube formation) and E18.5 [40], resulting in an important brain expansion especially during late pregnancy [41]. To provide a more refined measure of the growth effects of the protein restriction model, we examined the effects of diet on craniofacial shape and brain morphology. Previous studies using rats as an animal model, show that craniofacial shape can be affected by maternal nutritional stress [12, 13]. Particularly, specimens affected by this environmental perturbation displayed relatively shorter and wider face and neurocranium. A significant limitation of such studies is the focus on postnatal stages, while the changes induces in early ontogeny have not been explored yet. The results of this study will help to understand how maternal undernutrition differentially affects fetal, maternal and placental development and begin to reveal adaptive responses of the placenta.

## Materials and Methods

### Experimental design

C57BL/6J male and female 4-wk-old mice were purchased from The Jackson Laboratory (Bar Harbor, ME) and maintained on a 12-h light-12-h dark cycle for 4 weeks. Then, nulliparous females were randomly divided into two groups: Control (C), with *ad libitum* access to a 20% protein diet; and Low Protein (LP), with *ad libitum* access to a 6% protein diet (TD91352 and TD90016, Harlan Teklad, Madison). Both diets are isocaloric (3.8 Kcal/g) but differ in the amount of protein in the form of casein and DL-Methionine. No significant differences were found in the daily consumption of food among dams fed on control and low protein diets (*F*_1-63_ = 0.69; P = 0.41). The mice were bred after two weeks. Fetuses and placentas of at least 5 litters by conception day and treatment were dissected at embryonic days (E) 10.5, 17.5 and 18.5.

All animal care and procedures were conducted in the compliance with the University of Calgary Health Sciences Animal Care Committee and with the guidelines of the Canada Council on Animal Care (Protocol number M10076).

### Weight

Maternal, embryonic and placental weights were taken at the three time points. An ANOVA test was used to compare maternal weight in control and low protein groups before and after dissecting the uterus. Differences in embryo and placental size were tested using a mixed model ANOVA, in which maternal diet was the categorical predictor or fixed effect, while dams were included as a random factor. Due to high inter- and intra-litter variation in embryo development at E10.5, an univariate analysis of covariance (ANCOVA) including the number of tail somites as a covariate [42] was used in order to remove the variation due to development on feto-placental size.

### Cranial and brain size

Mouse heads were fixed overnight in 4% PFA. High resolution X-ray micro-computed tomography (μCT) images of the heads were acquired and 3D isosurfaces reconstructed as described in [43]. In addition, a subsample of specimens at E18.5 was re-scanned-using the same scanning protocol- to obtain images of the brain tissues. For this purpose, after 4% PFA immersion, the specimens were soaked overnight in Lugol’s iodine, which is used as a μCT contrast agent [44–46]. Briefly, 0.5 g of iodine and 1.5 g potassium iodide (KI) were mixed in 100 ml of water, heads were immerse in the iodine solution and keep at 4°C overnight. Then, the samples were rinsed in PBS to remove the excess of contrast agent and scanned.

A set of 42 bilateral anatomical points (landmarks) was digitized from the 3D reconstruction of each mouse cranium and a set of 57 landmarks were digitized on the μCT scans of the brain using Amira software (S1 Table). While the landmarks of the cranium were digitized on the reconstructed surfaces of the skull, a different approach was used for brain landmarking. First, a transversal plane that goes through the most anterior and posterior points of the brain was established. On this transversal slice 24 points were digitized, which mainly describe the relative length and width of the brains. Additionally, a parasagittal plane starting from the most anterior point of the left olfactory bulb was defined and 25 points were digitized on this view to capture variation in the height profile and in the antero-posterior dimensions as well (S1 Table).

The centroid size of the cranium and the brain were estimated as the square root of the summed squared distances from all landmarks to the centroid of each configuration [47]. While landmark-based methods have been widely used for estimating skull size, brain size has usually been assessed by its volume. In a μMRI sample of mouse brains, we had previously found that the centroid size (as measured here) and volume are highly correlated (0.928, p = 0.003) showing that the protocol used in this work is reliable to estimate brain size.

Size was compared between treatments at E17.5 and E18.5 by means of a mixed model ANOVA, using treatment as a fixed effect and dam as a random effect. A linear model was adjusted to the log transformed centroid sizes on body weight and the residuals from this model (i.e., deviations of actual size of each specimen from the value predicted by body weight) were compared by treatment and age. Positive values are expected if cranial and brain size are larger than expected for a given body weight. Head size was not estimated at E10.5 due to the difficulties of scanning young embryos.

### Craniofacial shape

All configurations of landmarks were translated to a common origin, scaled to the same size and rotated by minimizing the total sum of squared deviations of every landmark configuration from the mean configuration [47]. Differences among configurations that remain after this procedure represent actual shape differences. The effect of treatment and age on cranial shape was evaluated by means of a between-group principal component (bg-PCA) analysis. This method focuses on the effect of the specific factors to be tested [48] and thus is adequate to assess differences among groups in skull shape. In this analysis the superimposed coordinates of landmarks (i.e., shape variables) of all specimens were projected onto the eigenvectors of the between-groups covariance matrix. Shape changes along principal components were visualized by warping an average surface using the thin-plate spline procedure implemented by Landmark software [49]. Additionally, the variation in shape for each group was estimated as the mean of the squared Procrustes distances between each individual configuration from its group mean [50, 51]. This distance was estimated as the square root of the sum of squared distances between corresponding landmarks of each configuration and the group mean. To test whether the amount of variation differed among groups, we computed an ANOVA test of the individual deviations, using age and treatment as factors, which is an extension of Levene's test for multivariate data.

### Histological and in situ hybridization analysis of placentas

Placentas were weighed, fixed overnight in 4% phosphate buffered paraformaldehyde (PFA) and embedded in OCT (VWR) or dehydrated through ethanol and xylene gradients and embedded in paraffin. In situ hybridization using probes for *Tpbpa* and *Pcdh12* was performed as described previously [31].

For *in situ* hybridization, at least three central sections from at least three placentas (n = 3) were analyzed for each group and developmental stage. Placentas were bisected near the midpoint before dehydration and embedding and then at least one hundred 10 μm sections were taken for each placenta. From those sections, three central ones separated by 200 μm were chosen on the basis of the morphology: larger size and more conical shape of trophoblast for E10.5; larger size, presence of the umbilical cord vessels and/or central canal or spiral arteries for E17.5 and E18.5. The borders between three main placenta layers were distinguished by using the *Tpbpa* gene–marker of spongiotrophoblast and glycogen trophoblast cells in the junctional zone, and cell morphology. Glycogen trophoblast cells were distinguished by expression of *Pcdh12* mRNA. High magnification pictures were taken under the Leica microscope and then photomerged using a ‘Photomerge’ tool in the ‘Automate’ option of Photoshop software. Measurements of placenta layers areas and glycogen trophoblast cells areas were done using ImageJ software by manual outlining of the analyzed areas and the ‘polygon selection’ tool. Statistical significance was evaluated using two-tailed Student’s t-test assuming unequal variances. Additionally, a principal component analysis was performed using the areas of each placenta layer standardized by the total area to account for differences in size between ages. This multivariate analysis was used to reduce the dimensionality and summarise the variance in the dataset.

### Quantitative real-time PCR (qRT-PCR) for placental gene expression

From each litter, at least two specimens were dissected in order to isolate samples for RNA analysis. The corresponding placentas were immersed in 700 ml of Trizol, frozen and stored at -80°C until RNA extraction. The RNA was then processed using RNeasy Mini Kits (Qiagen) according to the manufacturer’s protocol. 1μg total RNA of three biological samples by experimental group was reverse-transcribed using the RT^2^ First Strand Kit (Qiagen). Complementary DNA samples produced were used for real time PCR to quantify the expression of several representative placenta-produced, prolactin-related genes (*Prl2c2*, *Prl3a1*, *Prl3b1*, *Prl3d1*, *Prl5a1*, *Prl7c1*, *Prl8a8*,), as well as spongiotrophoblast and glycogen trophoblast cell marker (*Tpbpa*), a glycogen trophoblast cell-specific marker (*Pcdh12*) and *Gapdh*. Quantitative RT-PCR analyses were performed with a DNA Engine Opticon 2 system (Bio-Rad Laboratories, Inc., MA, USA) using detection with SYBR Green I. Each sample was assessed in duplicate.

Real-Time qRT-PCR forward and reverse primers were obtained as QuantiTect Primer Assays or RT^2^ qPCR Primer Assays (Qiagen) for *Prl3d1* (QT01052219), *Prl5a1* (QT00139573), *Prl3a1* (QT00131782), *Prl8a8* (QT00124915), *Prl7c1* (QT00128646), *Pcdh12* (QT00100625), *GAPDH* (PPM02946E). Forward and reverse primers for *Prl2c2* were 5’-TGTGTGCAATGAGGAATGGT-3’ and 5’-TAGTGTGTGAGCCTGGCTTG-3’; for *Prl3b1* were 5’-CCACACTGCTGCAATCCTTA-3’ and 5’-TGACCATGCAGACCAGAAAG-3’, and for *Tpbpa* were 5’-AAGTTAGGCAACGAGCGAAA-3’ and 5’-AGTGCAGGATCCCACTTGTC-3’, respectively.

In order to determine the differences in target gene expression between control and low protein diet groups, relative standard curves based on series of 5 dilutions were obtained for each gene using an independent placenta sample from the control group. Each curve was done in triplicate and the average used as the reference. The ratio between the target and *Gapdh* expression was calculated to provide relative gene expression. Those normalized values were then used to estimate the fold-change between groups and compared by t tests.

## Results

### Placenta, embryo and maternal weight are reduced by low protein diet

**Fig 1:**
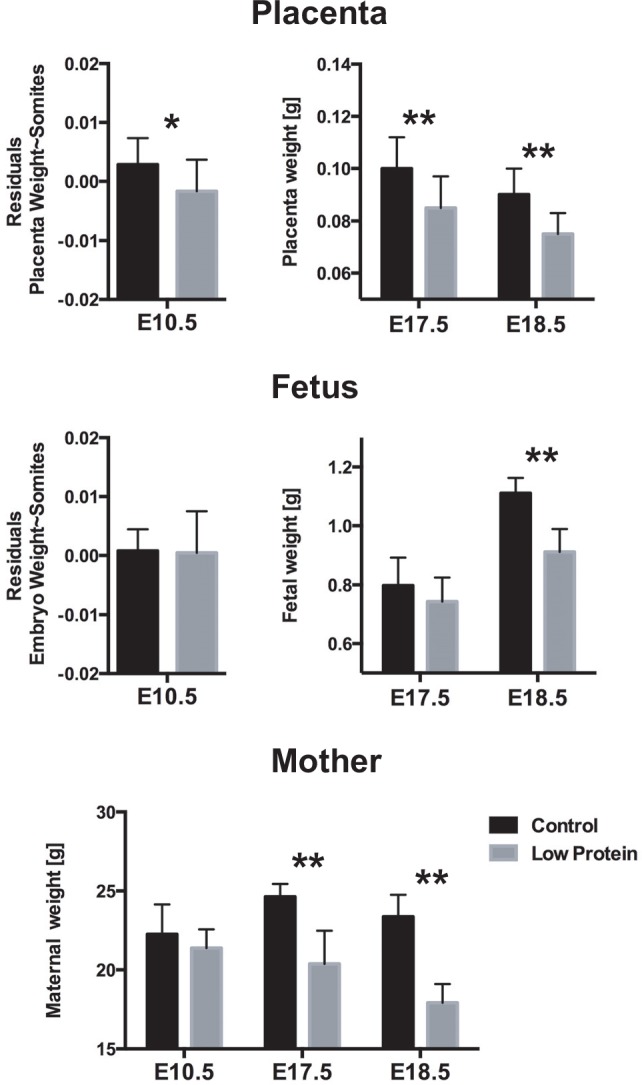
Maternal, placental and fetal weights at different developmental stages. E: embryonic day. The error bar represents the mean ± SD. *Different from control, P<0.051; **Different from control, P<0.01.

Nulliparous female mice were fed control or low protein diets for two weeks prior to mating with normal fed males. Time to mating and litter size estimated at E17.5 and E18.5 did not differ between experimental groups. The number of pups in the control group was 7.78±2.94 at E17.5 and 7.11±2.31 at E18.5, while in the low protein groups there were 7.5±2.12 and 8.42±1.61, respectively. Maternal weight was assessed after removal of the pregnant uterus and was not different between control and low protein groups at E10.5, but was significantly lower in the low protein group at E17.5 and E18.5 (Fig 1). Moreover, maternal weight of the low protein group significantly declined between E10.5 to E18.5 (P<0.001, assessed by two tailed t-test), while the weight of control mothers did not change significantly (Fig 1).

Embryo and placental weights were measured, and the number of tail somites was also assessed at E10.5 in order to account for potential differences in developmental stages. At E10.5, the effect of the number of tail somites was highly significant on both placenta and embryo weights (Table 1, Fig 1). After removing the effect of tail somites, there were no significant differences in embryo weight between control and low protein groups at E10.5 (Table 1, Fig 1). Fetal weight in the low protein group was slightly reduced, though not significantly, at E17.5 (*F*_1,13_ = 1.264, P = 0.281) but was 19% lower at E18.5 (*F*_1,13_ = 30.55, P<0.01) (Fig 1). In contrast to fetal growth, the low protein diet significantly reduced placenta weight as early as E10.5 (Table 1, Fig 1) and this persisted at E17.5 and E18.5, being 15% and 17% lighter than in control animals (*F*_1,13_ = 9.6, P<0.01; *F*_1,13_ = 25.5, P<0.01). The association between placental and fetal weight at each developmental stage, as measured by a Pearson correlation coefficient, was low at E17.5 (*r* = 0.45, P<0.01), but increased significantly by E18.5 (*r* = 0.69, P<0.01).

**Table 1:** ANCOVA test for placental and embryonic weight at E10.5.

Variable	Factors	F	p
**Placenta weight**	Tail somites	74.95	<0.0001
	Diet	7.25	0.02
	Tail somites*Diet	0.09	0.77
**Embryo weight**	Tail somites	248.49	<0.0001
	Diet	0.14	0.72
	Tail somites*Diet	0.21	0.65

### Fetal head size is only slightly reduced by low protein diet

**Fig 2:**
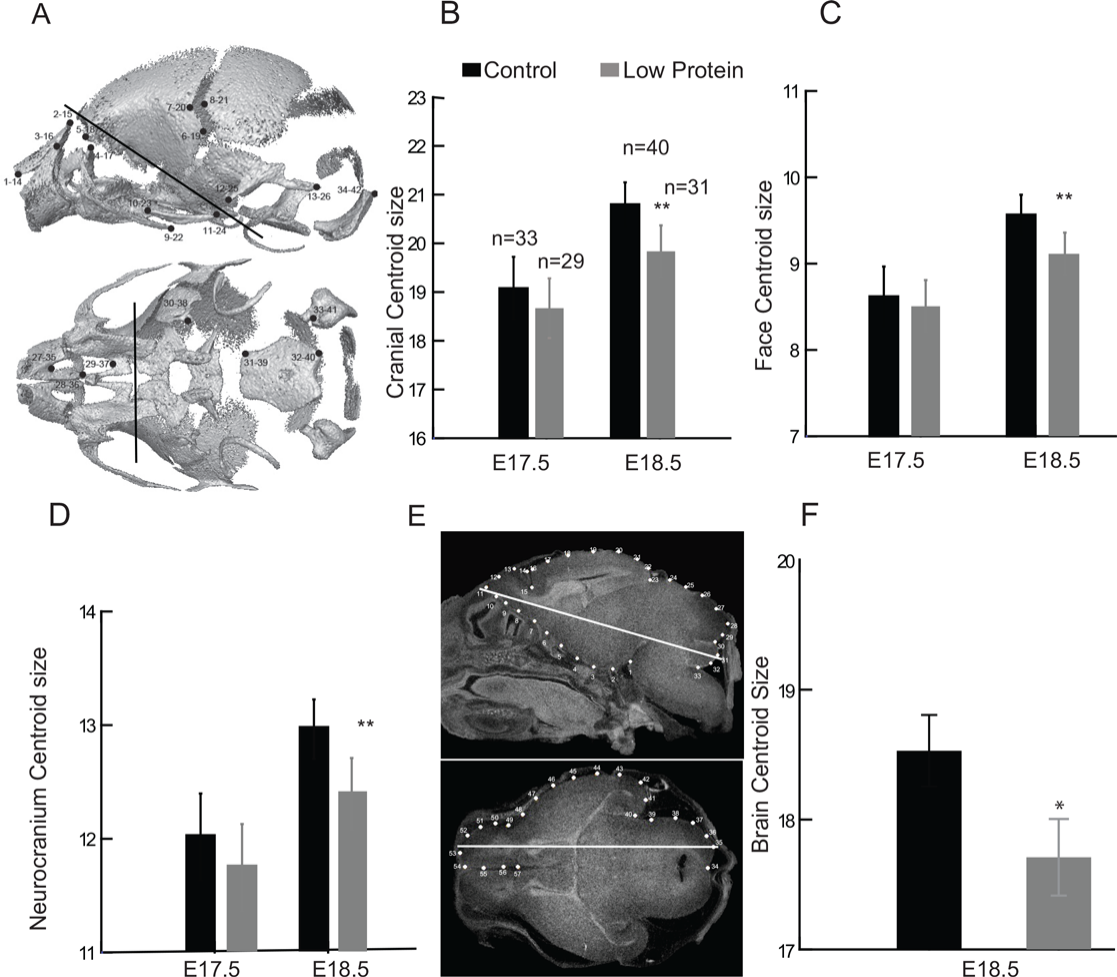
Changes in skull and brain size in the low protein fetuses. Coordinates of landmarks (A) and centroid size of the cranium (B), face (C) and neurocranium (D), by treatment and age. Units in μm. Coordinates of landmarks and semilandmarks digitized in sections of sagittal and axial planes of the brain (E). White lines represent the places where the transversal and sagittal sections were measured. Brain centroid size at E18.5 in the low protein and control groups (F). The error bar represents the mean ± SD. **Different from control, P<0.01. *Different from control, P<0.05.

At E17.5, cranial size was slightly (2.5% smaller) but not significantly different between control and low protein groups (*F*_1,9_ = 1.11, P = 0.32) (Fig 2B). Similar results were found for the face and neurocranium, which were 2.3% and 2.5% smaller in the low protein group (Fig 2C and 2D) but did not differ significantly from the control group (Face: *F*_1,9_ = 0.616, P = 0.453; Neurocranium: *F*_1,9_ = 1.26, P = 0.29). By E18.5, the overall cranial size as well as the size of face and neurocranium displayed drop of around 5% in the low protein group at E18.5 (Fig 2B, 2C and 2D). Significant differences were found between the low protein and control groups at this age (Cranium: *F*_1,13_ = 22.87, P<0.001; Face: *F*_1,13_ = 21.3, P< 0.001; Neurocranium: *F*_1,13_ = 24.11, P< 0.001).

**Fig 3:**
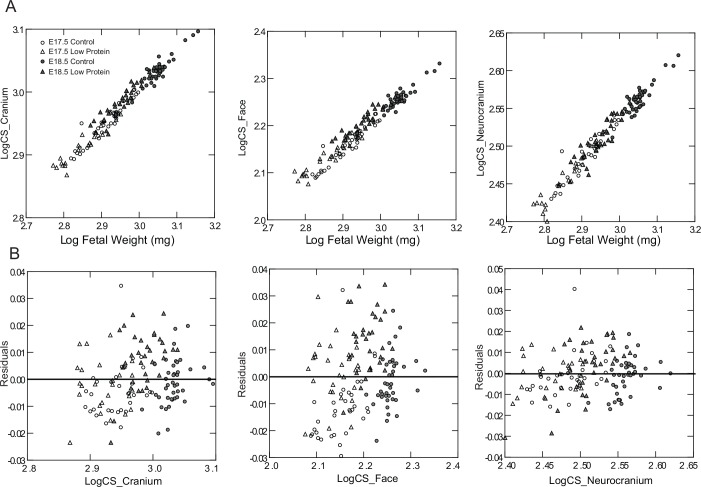
Relationship between fetal skull size and body weight in low protein and control groups. A) Linear regressions between log centroid size (CS) and log body weight (BW) for the cranium, face and neurocranium; B) residuals of linear regressions between log CS and BW. Triangles: low protein group; circles: control group; empty symbols: E17.5; filled symbols: E18.5.

Cranial size and fetal body weight displayed a strong linear relationship during the late period of fetal growth (Fig 3A), with the slopes of the regressions being 0.59 for the cranium, 0.66 for the face and 0.54 for the neurocranium. Head size adjusted for expected body size was higher in the low protein group compared to controls (Fig 3B). This indicates that individuals in the low protein groups have larger heads relative to body size than controls. Differences in the residuals between control and low protein groups were significant, particularly for the cranium and face (Cranium: *F*_1,129_ = 12.61, P<0.01; Face: *F*_1,129_ = 55.77, P<0.01; Neurocranium: *F*_1,129_ = 3.44, P = 0.06). The results obtained for cranial size are taken as proxies of brain growth. This is supported by the strong association between the centroid size of both structures evaluated at E18.5 (*r* = 0.699, P<0.01). Accordingly, there was a significant reduction in brain size by E18.5 in the low protein group (*F*_1,17_ = 4.52, P<0.05). The mean size of the brain was reduced by 4.5 per cent, a decrease similar in magnitude to that found for the neurocranium (Fig 2F).

### Cranial shape is affected by low protein diet

**Fig 4:**
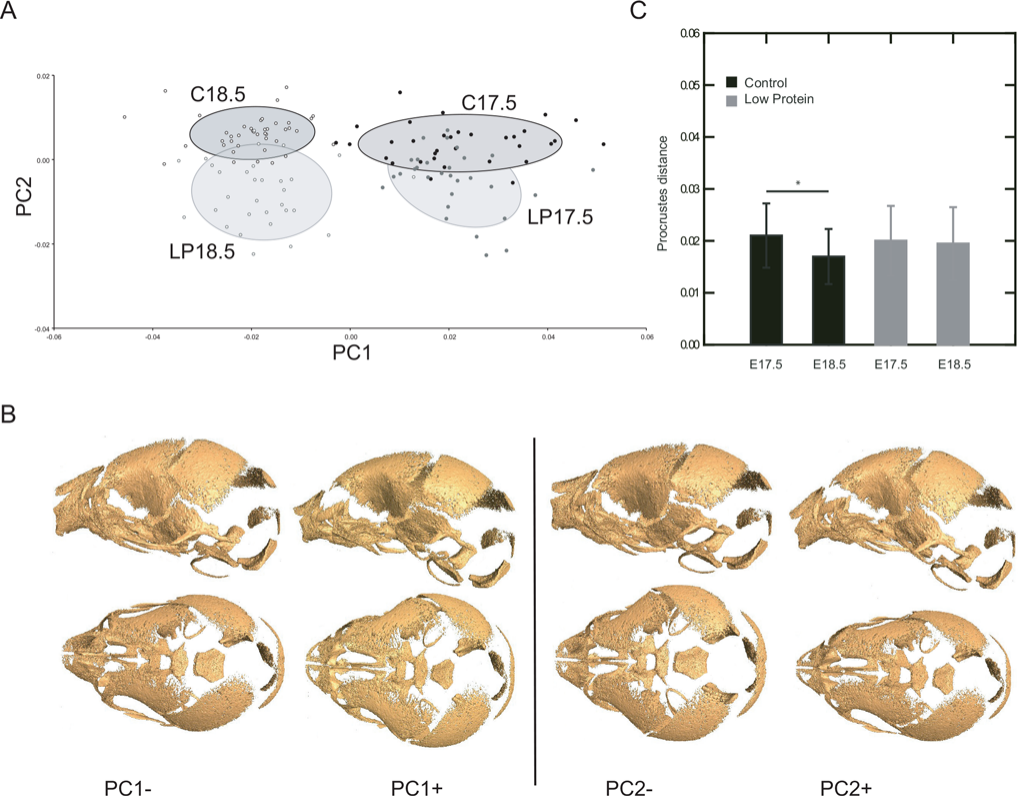
Analysis of skull shape based on three‐dimensional craniofacial landmarks. A) Distribution of specimens of low protein (LP) and control (C) groups along the between-group principal components (bg-PC). Ellipses represent the specimens of each group within the 1SD confidence interval. B) Shape changes corresponding to the observed extremes in the positive and negative directions of first two components shown as a warped surface of a mouse skull. C) Shape variance within each experimental group and age measured in units of Procrustes distance.

Variation in cranial shape was also assessed among treatments and age groups (Fig 4). The first principal component summarized shape variation related to fetal growth, given that E17.5 specimens were clearly separated from E18.5 along this axis (Fig 4A). Shape variation between age groups manifested primarily in the reduction of the relative width of the vault, the lengthening of the face and the less flexed cranial base at 18.5 days post-conception in relation to the cranial shape at 17.5 (Fig 4B). The second component accounted for differences in shape between treatments, which were characterized by wider and shorter skulls in the low protein groups compared to controls at both ages. These shape changes are shown as wireframes in S1 Fig. Interestingly, the amount of variation in shape between specimens was significantly reduced from E17.5 to E18.5 in the control group but not in the low protein group (Fig 4C).

### The junctional zone of the placenta is preferentially reduced by low protein diet

**Fig 5:**
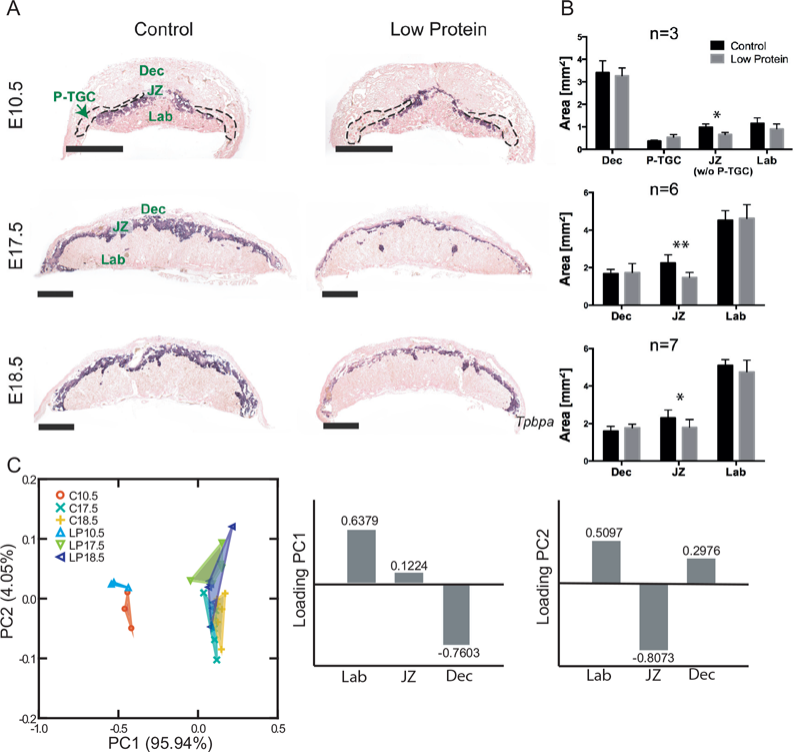
Decrease of junctional zone area in low protein placentas at E10.5, E17.5 and E18.5. A) In situ hybridization for *Tpbpa*, marker of junctional zone. Purple area: junctional zone; dashed area: parietal trophoblast giant cells (P-TGC); B) Bars represent average area of given placenta layer on a histological section ± SD. *Different from control, P<0.05; **Different from control, P<0.01; Dec: decidua; JZ: junctional zone; Lab: labyrinth; P-TGC: parietal trophoblast giant cells. Scale bar– 1 mm. C) Principal component analysis of the areas of the three main placenta layers for the control (C) and low protein groups (LP). The bars represent the loading of each variable on the first two principal components (PC).

Given that the first morphological evidence of impact of the low protein diet was on placental weight, well before maternal and fetal effects, we analyzed placenta structure and function in more detail. At all stages analyzed, the areas of the decidua and labyrinth were not different between low protein and control placentas. However, starting as early as E10.5, there was a significant reduction in the low protein group in the size of the junctional zone as defined by expression of the *Tpbpa* gene which is expressed in both spongiotrophoblast and glycogen trophoblast cells (Fig 5A and 5B). On histological sections, the reduction in cross-sectional areas was 33%, 34% and 22% at E10.5, 17.5 and 18.5 respectively. When the areas of the three main layers are analyzed together, as expected, a clear separation is seen between placentas at E10.5 and the other two ages (Fig 5C). Differences between treatments were also observable along the second principal component, although they were not as large as between age groups. The variable that contributed most to this component was the area of the junctional zone, which was relatively smaller in the specimens located at the positive values of the second component (the region where most specimens of the low protein groups are located).

**Fig 6:**
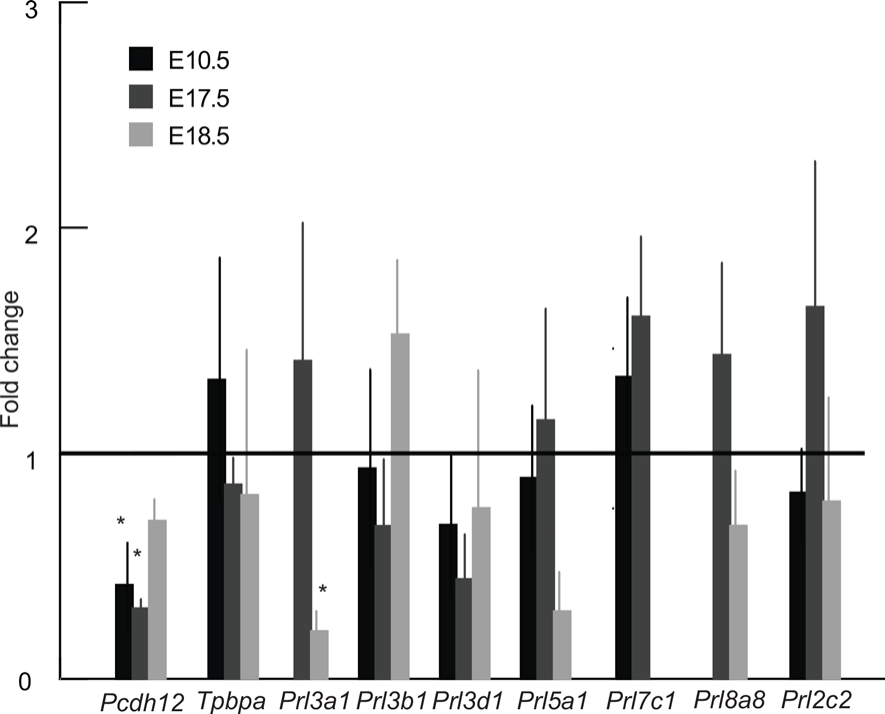
Expression of seven prolactin family genes, *Pcdh12* and *Tpbpa* in control and low protein placentas at E10.5, E17.5 and E18.5. Missing bars mean that the particular gene is not expressed at the given developmental stage. *Different from control, p<0.05. The error bar represents the mean ± SD. C–control group; LP–low protein group.

To further investigate the morphological changes in the junctional zone, quantitative real-time PCR assays were performed. Expression of *Pcdh12* mRNA, a marker of glycogen trophoblast cells, was significantly reduced in low protein placentas at E10.5 and E17.5 as compared to the control group, though there was no significant difference by E18.5 (Fig 6). By contrast, *Tpbpa* mRNA expression was not different suggesting that glycogen cells were specifically affected and not spongiotrophoblast cells. Based on in situ hybridization, the total area per section of *Pcdh12*-positive cells at E10.5 (0.53±0.04mm^2^ vs 0.32±0.13mm^2^) and E17.5 (0.33±0.08mm^2^ in control and 0.20±0.06mm^2^) tended to be reduced in the low protein group though the differences were not statistically significant. The parietal trophoblast giant cell component of the placenta did not seem to be affected by low protein diet at E10.5 (Fig 5A and 5B), E17.5 and E18.5 based on examination of histological sections. Consistent with this, expression of parietal trophoblast giant cell marker genes *Prl2c2*, *Prl3b1*, and *Prl3d1* based on qRT-PCR was not different between low protein and control group (Fig 6).

### Expression of Prolactin-related hormone genes

To determine if low protein diet induced compensatory changes in the endocrine function of the placenta, we assessed the expression of a subset of the placenta-specific, Prolactin-related hormones which have been implicated in regulation of metabolism and fetal growth [25, 26]. We focused on 7 of the 22 family members which are expressed in late gestation when fetal growth increases exponentially and representing at least one member from each of the six sub-families [26]: *Prl2c2*, *Prl3a1*, *Prl3b1*, *Prl3d1*, *Prl5a1*, *Prl7c1* and *Prl8a8*. Among these genes, only expression of *Prl3a1* was significantly different between the low protein and control groups. *Prl3a1* is expressed only by spongiotrophoblast cells in the junctional zone and levels are first detectable at E14.5 and increase towards term, paralleling the increase in fetal growth [52]. Interestingly, while *Prl3a1* mRNA levels seemed to be elevated in the low protein group at E17.5, expression declined and was significantly lower than control by E18.5, paralleling the arrest of fetal growth (Fig 6).

## Discussion

**Fig 7:**
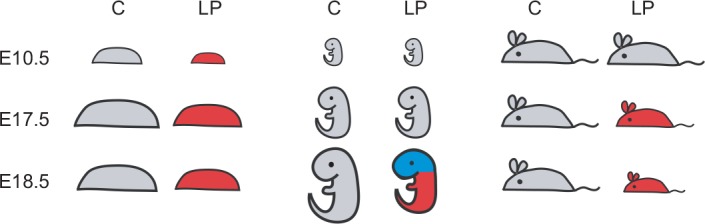
Schematic summary of the effects of low protein diet on placental, fetal and maternal weight and on the head size. C–control group; LP–low protein group; red color–weight significantly reduced as compared to the control group of the same age; blue color–cranial and brain size significantly different from the control of the same age.

In this study we demonstrate that the fetal, placental and maternal compartments are differentially affected by prolonged maternal protein restriction in mice. Overall, placenta size and function was first to be affected as a result of the low protein diet, as early as E10.5. The weight of the mother was next to be affected (E17.5), while the embryo weight did not display any significant change until the very end of the gestation (E18.5), and cranial size was relatively spared compared to the rest of the fetus (Fig 7). Further, we show that craniofacial shape is altered at E17.5 but to a greater extent at E18.5 in the low protein diet group. These differences in timing suggest that adaptations in the placenta and mother take place during early and mid pregnancy to maintain fetal growth, and especially the growth of brain. Other models for IUGR, such as *Igf2* knockout mice and rats, and mice fed on protein or calorie restricted diets, also show that fetal weight is not altered until the end of gestation [22, 23, 52, 53]. Nonetheless, most experiments have evaluated only one developmental stage, usually at E17.5 or later, and when more sampling points have been included, they have not started earlier than E14.5 [24, 54]. As our results point out, data on early gestation are essential to assess the processes that underlie a given outcome at a specific time point.

One phenomenon that has attracted considerable attention in association to IUGR in human neonates is that brain tissues are often affected to a lesser extent than body mass [14, 55]. We show here that even though normal fetal and brain growth cannot be maintained until the end of gestation under extended periods of maternal protein malnutrition, head size is larger than predicted by body size in the restricted group. This evidence of head-sparing is consistent with a preferential allocation of nutrients to brain tissues during pregnancy when the brain is expected to be especially demanding due to its high growth rate. With few exceptions, the majority of the animal research on perturbed growth of prenatal brain has been conducted in sheep models [56, 57]. Studies on rodents, while numerous, mainly focus on the negative effects of early environmental perturbations assessed postnatally [58]. Recently, a rising of cerebral blood velocity was observed in fetal mice exposed to acute hypoxia, which suggests active regulation of cerebral oxygenation and brain sparing in fetal mice [59]. This potential mechanism has not been tested under other stimuli such as nutrient restriction. However, our results are consistent with these findings and support the use of mice fed on low protein diets throughout pregnancy as a model to study head sparing.

In addition to brain and cranial size, nutrient restriction also altered cranial shape. This is consistent with earlier work that showed that both pre- and postnatal nutritional stress alters craniofacial shape [12, 60, 61]. This occurs primarily through the altered proportions of the brain, basicranium and face. If relative brain size is preserved but the rest of the skull is smaller, this results in an integrated series of changes that impact mid-facial projection, basicranial shape and the basicranial angle as well as the relative proportion of the neurocranium to the remainder of the skull [62–65].These are exactly the shape changes that we observed in our results. Therefore, these shape effects provide us with an additional indication of how the fetus is responding to the maternal malnutrition in our design. While we find detectable shape effects at E17.5, these are greatly exacerbated by E18.5 which is consistent with the interpretation that the fetus is spared during early gestation but that the effects become increasingly severe in late gestation.

The sequence of events in response to protein restriction suggests how fetal growth is spared. Protein restriction resulted in reduced placental size evident at early gestation (E10.5) and this reduction persisted until the end of pregnancy. Interestingly, chronic maternal protein restriction did not affect all placental layers to the same extent. The placenta layer most affected by low protein diet was the junctional zone, similar to previous results in undernourished mice [16, 18, 19], and specifically glycogen trophoblast cells. Since glycogen trophoblast cells accumulate and store glucose in the form of glycogen during gestation, we hypothesize that they act as the nutrient buffer between maternal supply and fetal demand, accumulating excess glucose from maternal blood in early and mid gestation, in order to utilize it in late gestation when the extra, easily accessible energy is needed for the rapid embryonic growth. We hypothesize that the reduction in glycogen trophoblast cells in placentas from undernourished pregnancies simply reflects the fact that glucose is limited. Between E10.5 and 17.5, though fetal growth was still normal, maternal (non-uterine) body weight was beginning to fall, indicating maternal “sacrifice” in favor of the growing fetus. It will be interesting to explore specific tissues and how they respond, and also to know what signals drive catabolism in the mother.

The placenta is an important endocrine organ, producing dozens of different hormones thought to regulate maternal adaptations and fetal responses to pregnancy. In mice, over half of the polypeptide hormones made by the placenta are encoded by 22 placenta-specific, Prolactin-related hormone genes. We observed a significant change in expression in response to low protein diet in only one of them, *Prl3a1*, which is expressed only in spongiotrophoblast cells of the junctional zone from E14.5 to term [66]. Its expression seemed to be elevated compared to controls at E17.5, but dramatically declined by E18.5 in the low protein fed group. *Prl3a1* function is unknown, though it is most closely related to *Prl3b1* and *Prl3d1* which work through the Prolactin receptor [34]. Other studies have shown that *Prl3a1* mRNA levels are significantly reduced at term in the placenta of feed restricted mice [67] and administration of the synthetic corticosteroid, dexamethasone, increases expression of *Prl3a1* in late gestation [68]. Overall these findings suggest a potential role for *Prl3a1* in normal late gestation for promotion of fetal growth, but this becomes dysregulated by term in response to low protein diet. What drives this change is unknown.

The strengths of this study lie in looking at maternal and fetal growth at multiple time points, including early pregnancy, and in analyzing changes in placenta under maternal malnutrition on cellular and hormonal levels. In humans there is evidence of decreased fetal and placental weight at term related to in utero nutrient compromise caused by events such as famine or fasting [69]. However, those studies report only the gross placental shape and size. It would be interesting to know which specific cell types in human placenta are particularly affected by in utero undernutrition and which placental hormones do not follow the normal temporal pattern of expression in such conditions. This is an important gap in knowledge to fill in order to understand the role of placenta in mediating nutritional challenges in human pregnancy. Human placenta-specific hormones related to Growth Hormone have been implicated in regulation of maternal metabolism and fetal growth [36] and are the most obvious candidates.

Evaluation of growth in human fetuses is usually done using ultrasound from which measurements such as femoral length and head circumference are obtained and then compared with published standards in order to identify growth retardation [70]. A small placenta size is known to be a cause of IUGR in humans and it has been shown that placenta volume predicts IUGR before fetal growth is compromised [71]. However, percentile curves for placental weight are rare [72–74]. Our study, although based on a mouse model, reinforces the importance of evaluating placental size for diagnostic purposes [75]. Early detection of retarded placental growth may allow improved prediction of IUGR before fetal growth is compromised along with the attendance of longer-term health consequences. These results highlight the interplay of placental development, placental buffering and fetal growth in determining birth outcomes in the face of human malnutrition.

In summary, this study demonstrates that the growth and function of the junctional zone of the murine placenta is affected by chronic maternal protein restriction in early gestation, and that reduction of this layer persists throughout gestation. However, fetal and especially brain growth are not affected until the very end of gestation. Based on these novel findings, we propose that the junctional zone is an early sensor of maternal nutritional status and that it initiates adaptive endocrine changes that mitigate the impact of poor maternal nutrition on fetal growth.

## Supporting Information

S1 Fig Shape changes corresponding to the observed extremes in the positive and negative directions of first two components. In the wireframes, darker lines represent shape changes relative to PC1 and PC2 score moving from both negative and positive directions, lighter lines represent the shape consensus. The upper panel shows the landmarks linked in the wireframes.
(PDF)

S1 Table Definition of landmarks digitized in the skull and the brain. Landmarks’ positions are illustrated in Fig 2.
(DOC)
