# Supplementary figures and images for: Chronic Protein Restriction in Mice Impacts Placental Function and Maternal Body Weight before Fetal Growth
Source: PLoS One. 2016 Mar 28;11(3):e0152227. doi: 10.1371/journal.pone.0152227 (PMC4809512; doi:10.1371/journal.pone.0152227)

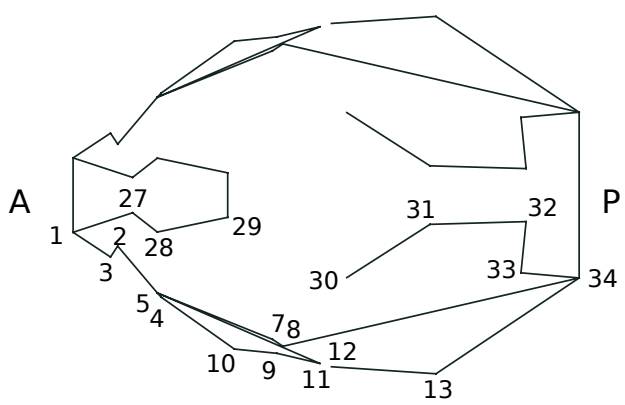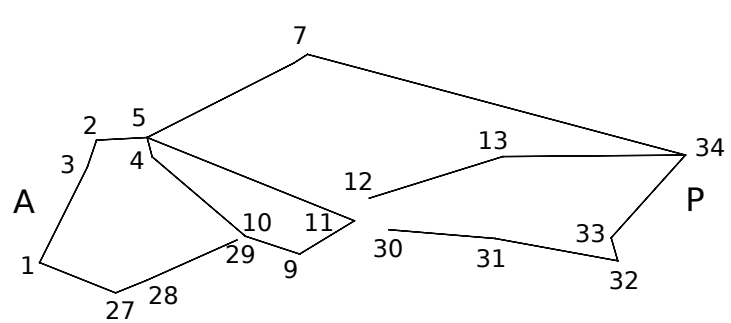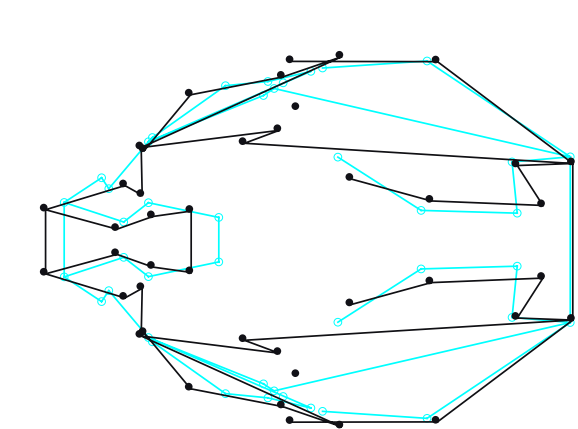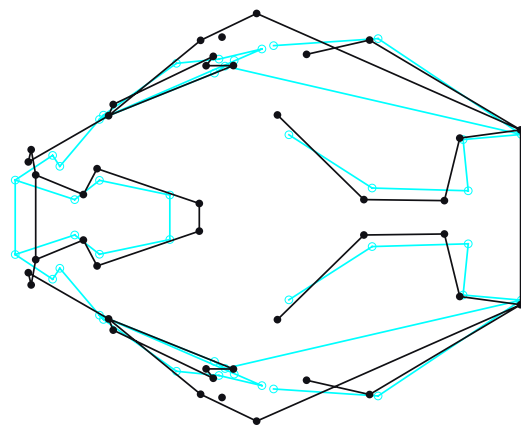

PC1-

PC1+

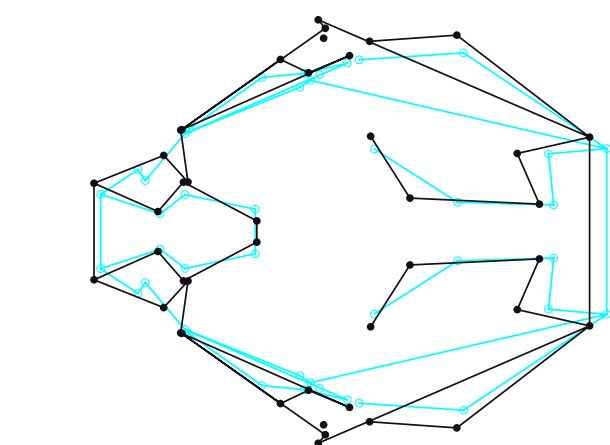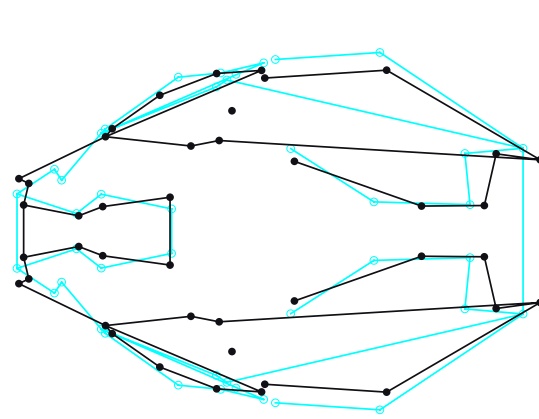

PC2-

PC2+

Supplement: S1 Fig — In the wireframes, darker lines represent shape changes relative to PC1 and PC2 score moving from both negative and positive directions, lighter lines represent the shape consensus. The upper panel shows the landmarks linked in the wireframes. (PDF) [file pone.0152227.s001.pdf]
