# Supplementary material for: Chronic Protein Restriction in Mice Impacts Placental Function and Maternal Body Weight before Fetal Growth
Source: PLoS One. 2016 Mar 28;11(3):e0152227. doi: 10.1371/journal.pone.0152227 (PMC4809512; doi:10.1371/journal.pone.0152227)
Supplement: S1 Table — Landmarks’ positions are illustrated in Fig 2. (DOC) [file pone.0152227.s002.doc]

**Supplementary Material**

Supplemental Table 1. Definition of landmarks digitized in lateral and ventral view of the fetal skull at E17.5 and E18.5.

| Number | Definition | View |
| --- | --- | --- |
| 1 | Most superior anterior point of the premaxilla, left side | Lat |
| 2 | Most posterior lateral point of the nasal bone, left side | Lat |
| 3 | Most superior posterior point of the premaxilla, left side | Lat |
| 4 | Intersection of frontal process of maxilla with frontal and lacrimal bones, left side | Lat |
| 5 | Most anterior lateral point of the frontal bone, taken close to the frontal process of maxilla, left side | Lat |
| 6 | Most lateral intersection of the frontal, parietal and squamous bones, taken on the frontal, left side | Lat |
| 7 | Most lateral intersection of the frontal and parietal bones, taken on the frontal, left side | Lat |
| 8 | Most lateral intersection of the frontal and parietal bones, taken on the parietal, left side | Lat |
| 9 | Intersection of zygoma process of maxilla, taken on maxilla, left side | Lat |
| 10 | Intersection of zygoma process of maxilla, taken on zygoma, left side (anterior) | Lat |
| 11 | Intersection of zygoma with zygomatic process of temporal, taken on zygoma, left side | Lat |
| 12 | Most anterior point on the anterior extension of the forming squamosal, left side | Lat |
| 13 | Most posterior point on the posterior extension of the forming squamosal, left side | Lat |
| 14 | Most superior anterior point of the premaxilla, right side | Lat |
| 15 | Most posterior lateral point of the nasal bone, right side | Lat |
| 16 | Most superior posterior point of the premaxilla, right side | Lat |
| 17 | Intersection of frontal process of maxilla with frontal and lacrimal bones, right side | Lat |
| 18 | Most anterior lateral point of the frontal bone, taken close to the frontal process of maxilla, right side | Lat |
| 19 | Most lateral intersection of the frontal, parietal and squamous bones, taken on the frontal, right side | Lat |
| 20 | Most lateral intersection of the frontal and parietal bones, taken on the frontal, right side | Lat |
| 21 | Most lateral intersection of the frontal and parietal bones, taken on the parietal, right side | Lat |
| 22 | Intersection of zygoma process of maxilla, taken on maxilla, right side | Lat |
| 23 | Intersection of zygoma process of maxilla, taken on zygoma, right side (anterior) | Lat |
| 24 | Intersection of zygoma with zygomatic process of temporal, taken on zygoma, right side | Lat |
| 25 | Most anterior point on the anterior extension of the forming squamosal, left side | Lat |
| 26 | Most posterior point on the posterior extension of the forming squamosal, right side | Lat |
| 27 | Most anterior point of the anterior palatine foramen, left side | Ventral |
| 28 | Most inferior-lateral point of the premaxillary-maxillary suture, taken on premaxilla, left side | Ventral |
| 29 | Most anterior point of the posterior palatine foramen, left side | Ventral |
| 30 | Posterior-medial point of the inferior portion of the left side of the aliesphenoid, left side | Internal |
| 31 | Most anterior-lateral point corner of the basioccipital, left side | Ventral |
| 32 | Most posterior-lateral point corner of the basioccipital, left side | Ventral |
| 33 | Point on the most medial point of the lateral occipital, in the hypoglossal canal, left side | Ventral |
| 34 | Superior posterior point on the ectocranial surface of occipital lateralis on the foramen magnum, left side | Lat |
| 35 | Most anterior point of the anterior palatine foramen, right side | Ventral |
| 36 | Most inferior-lateral point of the premaxillary-maxillary suture, taken on premaxilla, right side | Ventral |
| 37 | Most anterior point of the posterior palatine foramen, left side | Ventral |
| 38 | Posterior-medial point of the inferior portion of the left side of the aliesphenoid, right side | Internal |
| 39 | Most anterior-lateral point corner of the basioccipital, right side | Ventral |
| 40 | Most posterior-lateral point corner of the basioccipital, right side | Ventral |
| 41 | Point on the most medial point of the lateral occipital, in the hypoglossal canal, right side | Ventral |
| 42 | Superior posterior point on the ectocranial surface of occipital lateralis on the foramen magnum, right side | Lat |

Supplemental Table 2. Definition of landmarks digitized on fetal brains at E17.5 and E18.5..

| Number | Definition |
| --- | --- |
| 1 | Point located on the sulcus formed by the junction between the medulla and the hypothalamus |
| 2-10 | Semilandmarks along the inferior curve of the hypothalamus, the striatum septum and the olfactory bulb, between landmarks 1 and 11 |
| 11 | Most anterior point of the olfactory bulb |
| 12-14 | Semilandmarks along the olfactory bulb between landmarks 11 and 15 |
| 15 | Point located on the sulcus formed by the junction between the olfactory bulb and the cortex |
| 16-22 | Semilandmarks along the superior curve of the cortex |
| 23 | Point located on the junction between the cortex and the midbrain |
| 24-29 | Semilandmarks along the superior curve of the midbrain between the landmarks 23 and 30 |
| 30 | Point located on the junction between the midbrain and the cerebelum |
| 31-32 | Semilandmarks in the posterior curve of the cerebellum, between landmarks 30 and 33 |
| 33 | Point located on the posterior limit between the cerebellum and the medulla |
| 34 | Most posterior point of junction between right and left cerebellar halves, at the sagittal plane |
| 35-39 | Semilandmarks along posterior and lateral curve of the cerebellum, between landmarks 34 and 40 |
| 40 | Most posterior point on the junction between the cerebellum and the midbrain |
| 41-48 | Semilandmarks along the lateral curve of the midbrain and the cortex, between 40 and 49 |
| 49 | Most lateral point at the junction between the cortex and the olfactory bulb |
| 50-53 | Semilandmarks along the lateral and anterior curve of the olfactory bulb, between landmarks 49 and 54 |
| 54 | Most anterior point on the junction between the right and left parts of the olfactory bulb, at the sagittal plane |
| 55-56 | Semilandmarks along the limit between the right and left parts of the olfactory bulb, between landmarks 54 and 57 |
| 57 | Most posterior point in the junction between the right and left parts of the olfactory bulb, at the sagittal plane |
